# 2,4,6,8-Tetra­kis(4-fluoro­phen­yl)-3,7-diaza­bicyclo­[3.3.1]nonan-9-one

**DOI:** 10.1107/S1600536808039135

**Published:** 2008-11-29

**Authors:** S. Natarajan, V. Sudhapriya, V. Vijayakumar, N. Shoba, J. Suresh, P. L. Nilantha Lakshman

**Affiliations:** a Department of Physics, Madurai Kamaraj University, Madurai 625 021, India; b Department of Chemistry, VIT University, Vellore 632 014, India; c Department of Physics, The Madura College, Madurai 625 011, India; d Department of Food Science and Technology, Faculty of Agriculture, University of Ruhuna, Mapalana, Kamburupitiya 81100, Sri Lanka

## Abstract

In the title compound, C_31_H_24_F_4_N_2_O, the bicyclo­[3.3.1]nonane ring exists in a chair-boat conformation. Two of the four fluorine-substituted rings adopt equatorial dispositions with the piperidin-4-one rings. Mol­ecules are linked into a two-dimensional network parallel to (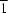
01) by N—H⋯O, C—H⋯F and C—H⋯O hydrogen bonds. Inter­molecular N—H⋯π and C—H⋯π inter­actions are also observed.

## Related literature

For general background, see: Asakawa (1995 ▶); Jeyaraman & Avila (1981 ▶).
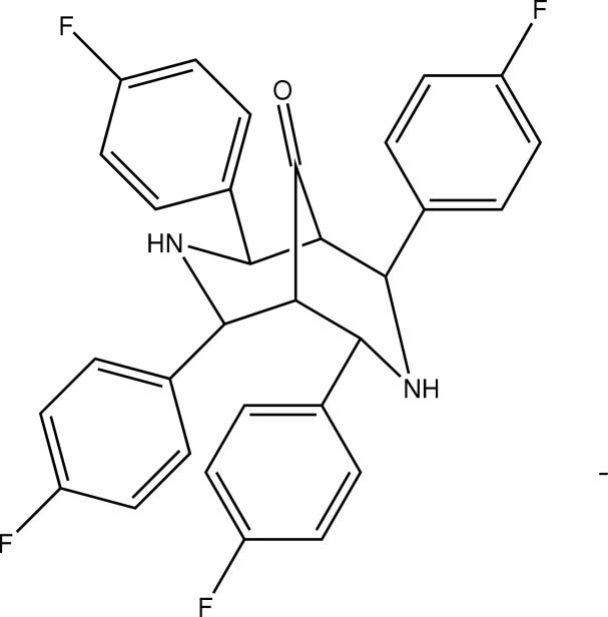


## Experimental

### 

#### Crystal data

C_31_H_24_F_4_N_2_O
*M* *_r_* = 516.52
Monoclinic, 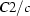

*a* = 37.1521 (9) Å
*b* = 7.1458 (5) Å
*c* = 26.2165 (7) Å
β = 133.249 (4)°
*V* = 5069.5 (4) Å^3^
*Z* = 8
Mo *K*α radiation
μ = 0.10 mm^−1^
*T* = 293 (2) K
0.19 × 0.16 × 0.11 mm

#### Data collection

Nonius MACH-3 diffractometer
Absorption correction: ψ scan (North *et al.*, 1968 ▶) *T*_min_ = 0.986, *T*_max_ = 0.991
5315 measured reflections
4465 independent reflections
2735 reflections with *I* > 2σ(*I*)
*R*_int_ = 0.024
2 standard reflections frequency: 60 min intensity decay: none

#### Refinement

*R*[*F* ^2^ > 2σ(*F* ^2^)] = 0.039
*wR*(*F* ^2^) = 0.112
*S* = 1.02
4465 reflections
351 parameters
H atoms treated by a mixture of independent and constrained refinement
Δρ_max_ = 0.14 e Å^−3^
Δρ_min_ = −0.23 e Å^−3^

### 

Data collection: *CAD-4 EXPRESS* (Enraf–Nonius, 1994 ▶); cell refinement: *CAD-4 EXPRESS*; data reduction: *XCAD4* (Harms & Wocadlo, 1996 ▶); program(s) used to solve structure: *SHELXS97* (Sheldrick, 2008 ▶); program(s) used to refine structure: *SHELXL97* (Sheldrick, 2008 ▶); molecular graphics: *PLATON* (Spek, 2003 ▶); software used to prepare material for publication: *SHELXL97*.

## Supplementary Material

Crystal structure: contains datablocks global, I. DOI: 10.1107/S1600536808039135/ci2727sup1.cif

Structure factors: contains datablocks I. DOI: 10.1107/S1600536808039135/ci2727Isup2.hkl

Additional supplementary materials:  crystallographic information; 3D view; checkCIF report

## Figures and Tables

**Table 1:** Hydrogen-bond geometry (Å, °)

*D*—H⋯*A*	*D*—H	H⋯*A*	*D*⋯*A*	*D*—H⋯*A*
C15—H15⋯F4^i^	0.93	2.52	3.254 (3)	136
C3—H3⋯O1^ii^	0.98	2.56	3.358 (2)	138
N2—H1*A*⋯O1^iii^	0.86 (2)	2.53 (2)	3.292 (2)	148 (3)
N1—H2*A*⋯*Cg*3^iv^	0.89 (2)	2.70 (3)	3.549 (3)	160 (2)
C36—H36⋯*Cg*2^v^	0.93	2.81	3.696 (3)	160
C42—H42⋯*Cg*1^v^	0.93	2.78	3.651 (3)	157
C45—H45⋯*Cg*3^iii^	0.93	2.65	3.494 (3)	151

Symmetry codes: (i) 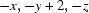; (ii) 
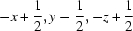
; (iii) 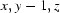; (iv) 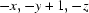; (v) 
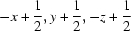
. *Cg*1, *Cg*2 and *Cg*3 are the centroids of the C31–C36, C41–C46 and C61–C66 rings, respectively.

**Fig. 1.**
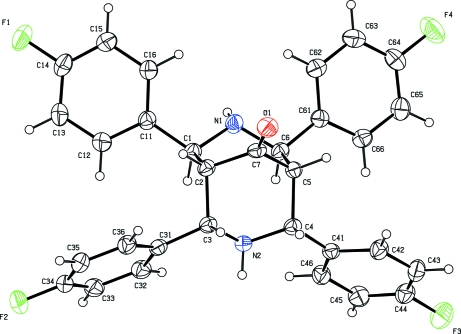
The molecular structure of the title compound, showing 50% probability displacement ellipsoids and the atom-numbering scheme.

**Fig. 2.**
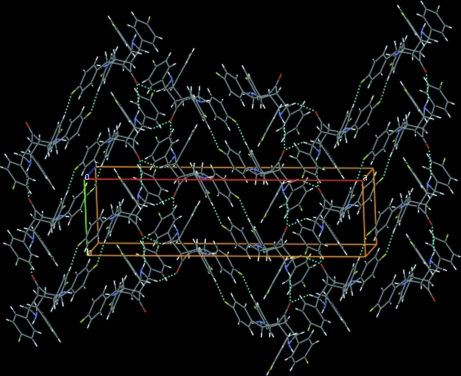
Packing diagram viewed down the c axis.

## supplementary crystallographic information

### Comment

Azabicyclononane and their derivatives are studied intensively because of their pharmaceutical use and their application as an important structure in the field of molecular recognition. The 3-azabicyclo[3.3.1] nonane skeletal system easily constructed *via* a double Mannich reaction (Jeyaraman & Avila, 1981), has been known for some time. The bicyclo[3.3.1]nonane carbon framework is frequently encountered in natural products, in particular in alkaloids and terpenoids, *e.g.* trifarienols (Asakawa, 1995). Further, the study of conformation of the bicyclic ring helps in the understanding of interactions that are possible between the substituted aryl rings.

The molecular structure of the title compound is shown in Fig.1. The bicyclic [3.3.1]nonane ring can exist in chair-chair, chair-boat and boat-boat conformations. Among these, the chair-chair conformation is the most favourable. In the title compound, the bicyclic ring system adopts a chair-boat conformation. In the N1-piperidine ring of the compound, atoms N1 and C7 deviate from the C1/C2/C5/C6 plane by 0.652 (3) and 0.685 (3) Å, respectively, indicating a nearly ideal boat conformation. The phenyl rings substituted at C1 and C6  positions are oriented at an angle of 28.2 (1)° to each other. The phenyl rings substituted at C3 and C4 are oriented with an angle of 28.6 (1)° between them and they are equatorially disposed with respect to the piperidine ring, with torsion angles C7—C5—C4—C41 = -175.1 (2)° and C7—C2—C3—C31 = 173.3 (2)°.

Fig. 2 shows the packing viewed down the *c* axis. Pairs of intermolecular C—H···F (Table 1) hydrogen bonds form centrosymmetric *R*_2_^2^(24) dimers. The moelcules are linked into a two-dimensional network parallel to the (101) by N—H···O, C—H···F and C—H···O hydrogen bonds. In addition, some C—H···π interactions (Table 1 ; Cg1, Cg2 and Cg3 refer to centroids of C31-C36, C41-C46 and C61-C66 rings, respectively).

### Experimental

A mixture of 0.73 ml of dry acetone (0.01 mol), 4.96 ml of 4-fluorobenzaldehyde (0.04 mol), 1.54 g dry ammonium acetate (0.02 mol) were taken in a flask with ethanol as solvent. Contents were heated with constant shaking until it becomes pale orange in colour. Then the contents were kept aside for 24 h and the title compound was filtered through the Buchner funnel, washed with 1:1 ethanol-ether mixture until the yellow colour disappeared and dried (yield 45%, m.p. 484 K).

### Refinement

Atoms H1A and H2A were located in a difference Fourier map and their positional and isotropic displacement parameters were refined. The remaining H atoms were placed in calculated positions and allowed to ride on their carrier atoms with C-H = 0.93–0.98 Å and *U*_iso_ = 1.2*U*_eq_(C) for CH group.

### Crystal data

**Table:**

C_31_H_24_F_4_N_2_O	*F*_000_ = 2144
*M**_r_* = 516.52	*D*_x_ = 1.353 Mg m^−^^3^
Monoclinic, *C*2/*c*	Mo *K*α radiation λ = 0.71073 Å
Hall symbol: -C 2yc	Cell parameters from 25 reflections
*a* = 37.1521 (9) Å	θ = 2–25º
*b* = 7.1458 (5) Å	µ = 0.10 mm^−^^1^
*c* = 26.2165 (7) Å	*T* = 293 (2) K
β = 133.249 (4)º	Block, colourless
*V* = 5069.5 (4) Å^3^	0.19 × 0.16 × 0.11 mm
*Z* = 8	

### Data collection

**Table:**

Nonius MACH-3 diffractometer	*R*_int_ = 0.024
Radiation source: fine-focus sealed tube	θ_max_ = 25.0º
Monochromator: graphite	θ_min_ = 2.1º
*T* = 293(2) K	*h* = 0→44
ω–2θ scans	*k* = −1→8
Absorption correction: ψ scan(North *et al.*, 1968)	*l* = −31→22
*T*_min_ = 0.986, *T*_max_ = 0.991	2 standard reflections
5315 measured reflections	every 60 min
4465 independent reflections	intensity decay: none
2735 reflections with *I* > 2σ(*I*)	

### Refinement

**Table:**

Refinement on *F*^2^	Secondary atom site location: difference Fourier map
Least-squares matrix: full	Hydrogen site location: inferred from neighbouring sites
*R*[*F*^2^ > 2σ(*F*^2^)] = 0.039	H atoms treated by a mixture of independent and constrained refinement
*wR*(*F*^2^) = 0.112	*w* = 1/[σ^2^(*F*_o_^2^) + (0.0552*P*)^2^ + 1.3466*P*] where *P* = (*F*_o_^2^ + 2*F*_c_^2^)/3
*S* = 1.02	(Δ/σ)_max_ = 0.001
4465 reflections	Δρ_max_ = 0.14 e Å^−^^3^
351 parameters	Δρ_min_ = −0.23 e Å^−^^3^
Primary atom site location: structure-invariant direct methods	Extinction correction: none

### Special details

**Table:**

Geometry. All e.s.d.'s (except the e.s.d. in the dihedral angle between two l.s. planes) are estimated using the full covariance matrix. The cell e.s.d.'s are taken into account individually in the estimation of e.s.d.'s in distances, angles and torsion angles; correlations between e.s.d.'s in cell parameters are only used when they are defined by crystal symmetry. An approximate (isotropic) treatment of cell e.s.d.'s is used for estimating e.s.d.'s involving l.s. planes.
Refinement. Refinement of *F*^2^ against ALL reflections. The weighted *R*-factor *wR* and goodness of fit *S* are based on *F*^2^, conventional *R*-factors *R* are based on *F*, with *F* set to zero for negative *F*^2^. The threshold expression of *F*^2^ > σ(*F*^2^) is used only for calculating *R*-factors(gt) *etc*. and is not relevant to the choice of reflections for refinement. *R*-factors based on *F*^2^ are statistically about twice as large as those based on *F*, and *R*- factors based on ALL data will be even larger.

### Fractional atomic coordinates and isotropic or equivalent isotropic displacement parameters (Å^2^)

**Table:**

	*x*	*y*	*z*	*U*_iso_*/*U*_eq_	
C1	0.11845 (6)	0.4616 (3)	0.20424 (9)	0.0401 (5)	
H1	0.1151	0.3251	0.2002	0.048*	
C2	0.17256 (7)	0.5147 (3)	0.24318 (10)	0.0385 (4)	
H2	0.1871	0.5885	0.2850	0.046*	
C3	0.20558 (6)	0.3394 (3)	0.26425 (9)	0.0378 (5)	
H3	0.2379	0.3838	0.2838	0.045*	
C4	0.18263 (7)	0.3426 (3)	0.15308 (9)	0.0377 (4)	
H4	0.2166	0.3848	0.1798	0.045*	
C5	0.14991 (6)	0.5202 (3)	0.12939 (9)	0.0371 (4)	
H5	0.1508	0.5974	0.0994	0.044*	
C6	0.09512 (6)	0.4758 (3)	0.09015 (9)	0.0398 (5)	
H6	0.0897	0.3403	0.0836	0.048*	
C7	0.17300 (7)	0.6262 (3)	0.19522 (10)	0.0386 (5)	
C11	0.10312 (7)	0.5332 (3)	0.24153 (10)	0.0430 (5)	
C12	0.11071 (9)	0.4228 (4)	0.29154 (12)	0.0621 (6)	
H12	0.1258	0.3067	0.3025	0.074*	
C13	0.09628 (10)	0.4816 (4)	0.32551 (13)	0.0711 (7)	
H13	0.1017	0.4068	0.3593	0.085*	
C14	0.07391 (8)	0.6514 (4)	0.30841 (12)	0.0571 (6)	
C15	0.06615 (8)	0.7656 (3)	0.26035 (12)	0.0564 (6)	
H15	0.0512	0.8817	0.2500	0.068*	
C16	0.08102 (8)	0.7055 (3)	0.22704 (11)	0.0504 (5)	
H16	0.0760	0.7829	0.1941	0.060*	
C31	0.21368 (7)	0.2239 (3)	0.31941 (9)	0.0394 (5)	
C32	0.18761 (7)	0.0614 (3)	0.30531 (11)	0.0476 (5)	
H32	0.1646	0.0158	0.2597	0.057*	
C33	0.19552 (8)	−0.0333 (3)	0.35830 (12)	0.0531 (6)	
H33	0.1778	−0.1413	0.3486	0.064*	
C34	0.22973 (8)	0.0344 (3)	0.42487 (11)	0.0520 (6)	
C35	0.25679 (8)	0.1928 (3)	0.44138 (11)	0.0526 (6)	
H35	0.2801	0.2361	0.4873	0.063*	
C36	0.24843 (7)	0.2856 (3)	0.38825 (10)	0.0462 (5)	
H36	0.2666	0.3929	0.3987	0.055*	
C41	0.16646 (6)	0.2296 (3)	0.09174 (9)	0.0377 (4)	
C42	0.17840 (7)	0.2953 (3)	0.05514 (10)	0.0486 (5)	
H42	0.1964	0.4056	0.0697	0.058*	
C43	0.16441 (8)	0.2022 (3)	−0.00210 (11)	0.0572 (6)	
H43	0.1725	0.2483	−0.0263	0.069*	
C44	0.13822 (8)	0.0401 (3)	−0.02224 (11)	0.0552 (6)	
C45	0.12559 (8)	−0.0312 (3)	0.01184 (11)	0.0550 (6)	
H45	0.1078	−0.1423	−0.0031	0.066*	
C46	0.13961 (7)	0.0644 (3)	0.06916 (10)	0.0476 (5)	
H46	0.1310	0.0176	0.0927	0.057*	
C61	0.06009 (7)	0.5723 (3)	0.01948 (10)	0.0406 (5)	
C62	0.03742 (8)	0.7397 (3)	0.00973 (12)	0.0590 (6)	
H62	0.0420	0.7904	0.0465	0.071*	
C63	0.00812 (9)	0.8329 (4)	−0.05342 (13)	0.0697 (7)	
H63	−0.0071	0.9450	−0.0595	0.084*	
C64	0.00196 (8)	0.7583 (4)	−0.10652 (11)	0.0596 (6)	
C65	0.02287 (8)	0.5928 (3)	−0.10022 (11)	0.0546 (6)	
H65	0.0177	0.5435	−0.1376	0.066*	
C66	0.05205 (7)	0.4997 (3)	−0.03665 (10)	0.0469 (5)	
H66	0.0665	0.3863	−0.0315	0.056*	
N1	0.08615 (6)	0.5436 (3)	0.13358 (8)	0.0448 (4)	
N2	0.18264 (6)	0.2354 (2)	0.20033 (8)	0.0396 (4)	
O1	0.19350 (5)	0.7766 (2)	0.21027 (7)	0.0521 (4)	
F1	0.05822 (5)	0.7074 (2)	0.34005 (7)	0.0799 (4)	
F2	0.23818 (6)	−0.0598 (2)	0.47714 (7)	0.0775 (4)	
F3	0.12520 (6)	−0.0562 (2)	−0.07769 (7)	0.0844 (5)	
F4	−0.02642 (6)	0.8518 (2)	−0.16843 (7)	0.0923 (5)	
H1A	0.1975 (8)	0.130 (3)	0.2109 (11)	0.057 (7)*	
H2A	0.0545 (8)	0.522 (3)	0.1100 (10)	0.052 (6)*	

### Atomic displacement parameters (Å^2^)

**Table:**

	*U*^11^	*U*^22^	*U*^33^	*U*^12^	*U*^13^	*U*^23^
C1	0.0412 (10)	0.0390 (12)	0.0455 (11)	0.0006 (9)	0.0318 (9)	0.0010 (9)
C2	0.0420 (10)	0.0344 (11)	0.0438 (10)	−0.0014 (9)	0.0313 (9)	−0.0037 (9)
C3	0.0366 (10)	0.0367 (11)	0.0427 (11)	0.0005 (9)	0.0282 (9)	−0.0008 (9)
C4	0.0358 (9)	0.0366 (11)	0.0442 (11)	−0.0001 (9)	0.0288 (9)	−0.0007 (9)
C5	0.0401 (10)	0.0341 (11)	0.0429 (10)	0.0006 (8)	0.0308 (9)	0.0023 (9)
C6	0.0395 (10)	0.0392 (12)	0.0443 (10)	−0.0017 (9)	0.0302 (9)	−0.0026 (9)
C7	0.0359 (10)	0.0317 (11)	0.0527 (12)	0.0042 (9)	0.0321 (9)	0.0020 (9)
C11	0.0399 (10)	0.0480 (13)	0.0455 (11)	−0.0007 (10)	0.0309 (9)	0.0007 (10)
C12	0.0749 (15)	0.0619 (16)	0.0706 (15)	0.0213 (13)	0.0580 (14)	0.0195 (13)
C13	0.0894 (18)	0.083 (2)	0.0738 (16)	0.0217 (16)	0.0686 (15)	0.0244 (15)
C14	0.0563 (13)	0.0735 (17)	0.0589 (14)	−0.0002 (12)	0.0463 (12)	−0.0053 (13)
C15	0.0618 (13)	0.0542 (15)	0.0660 (14)	0.0085 (12)	0.0487 (12)	0.0040 (12)
C16	0.0588 (13)	0.0490 (13)	0.0573 (12)	0.0035 (11)	0.0452 (11)	0.0051 (11)
C31	0.0411 (10)	0.0378 (11)	0.0454 (11)	0.0036 (9)	0.0320 (9)	−0.0010 (9)
C32	0.0539 (12)	0.0384 (12)	0.0529 (12)	−0.0034 (10)	0.0374 (11)	−0.0054 (10)
C33	0.0688 (14)	0.0370 (12)	0.0703 (15)	0.0002 (11)	0.0541 (13)	0.0017 (11)
C34	0.0722 (15)	0.0462 (14)	0.0604 (14)	0.0155 (12)	0.0543 (13)	0.0130 (12)
C35	0.0642 (14)	0.0536 (14)	0.0472 (12)	0.0036 (12)	0.0410 (12)	−0.0026 (11)
C36	0.0526 (11)	0.0424 (12)	0.0481 (11)	−0.0034 (10)	0.0362 (10)	−0.0050 (10)
C41	0.0370 (9)	0.0359 (11)	0.0425 (10)	0.0037 (9)	0.0282 (9)	0.0026 (9)
C42	0.0570 (12)	0.0449 (12)	0.0589 (13)	−0.0065 (10)	0.0455 (11)	−0.0042 (11)
C43	0.0716 (14)	0.0621 (16)	0.0597 (13)	−0.0006 (13)	0.0535 (13)	−0.0007 (12)
C44	0.0622 (13)	0.0568 (15)	0.0434 (11)	0.0048 (12)	0.0350 (11)	−0.0062 (11)
C45	0.0626 (13)	0.0443 (13)	0.0531 (12)	−0.0088 (11)	0.0378 (11)	−0.0084 (11)
C46	0.0554 (12)	0.0427 (13)	0.0520 (12)	−0.0045 (11)	0.0397 (11)	−0.0012 (10)
C61	0.0379 (10)	0.0403 (12)	0.0447 (11)	−0.0010 (9)	0.0288 (9)	0.0001 (9)
C62	0.0704 (15)	0.0555 (15)	0.0572 (13)	0.0157 (13)	0.0462 (13)	0.0046 (12)
C63	0.0783 (17)	0.0603 (17)	0.0675 (16)	0.0270 (14)	0.0488 (14)	0.0153 (14)
C64	0.0550 (13)	0.0605 (16)	0.0488 (13)	0.0061 (12)	0.0300 (11)	0.0138 (12)
C65	0.0497 (12)	0.0654 (16)	0.0438 (12)	−0.0071 (12)	0.0301 (11)	−0.0051 (12)
C66	0.0432 (11)	0.0477 (13)	0.0474 (12)	0.0013 (10)	0.0301 (10)	−0.0027 (10)
N1	0.0374 (9)	0.0567 (12)	0.0455 (9)	0.0043 (9)	0.0303 (8)	0.0037 (9)
N2	0.0479 (9)	0.0329 (10)	0.0443 (9)	0.0049 (8)	0.0340 (8)	0.0024 (8)
O1	0.0620 (9)	0.0362 (8)	0.0657 (9)	−0.0095 (7)	0.0467 (8)	−0.0059 (7)
F1	0.0926 (10)	0.1006 (12)	0.0882 (10)	0.0073 (9)	0.0781 (9)	−0.0025 (9)
F2	0.1203 (12)	0.0641 (9)	0.0821 (9)	0.0118 (8)	0.0825 (10)	0.0192 (8)
F3	0.1090 (11)	0.0841 (11)	0.0633 (8)	−0.0052 (9)	0.0602 (9)	−0.0224 (8)
F4	0.0987 (11)	0.0894 (12)	0.0591 (8)	0.0184 (9)	0.0426 (9)	0.0273 (8)

### Geometric parameters (Å, °)

**Table:**

C1—N1	1.473 (2)	C32—C33	1.384 (3)
C1—C11	1.518 (3)	C32—H32	0.93
C1—C2	1.554 (2)	C33—C34	1.362 (3)
C1—H1	0.98	C33—H33	0.93
C2—C7	1.498 (3)	C34—F2	1.356 (2)
C2—C3	1.566 (3)	C34—C35	1.372 (3)
C2—H2	0.98	C35—C36	1.372 (3)
C3—N2	1.459 (2)	C35—H35	0.93
C3—C31	1.505 (3)	C36—H36	0.93
C3—H3	0.98	C41—C42	1.386 (3)
C4—N2	1.456 (2)	C41—C46	1.389 (3)
C4—C41	1.508 (3)	C42—C43	1.377 (3)
C4—C5	1.562 (3)	C42—H42	0.93
C4—H4	0.98	C43—C44	1.365 (3)
C5—C7	1.503 (3)	C43—H43	0.93
C5—C6	1.559 (2)	C44—C45	1.361 (3)
C5—H5	0.98	C44—F3	1.363 (2)
C6—N1	1.470 (2)	C45—C46	1.387 (3)
C6—C61	1.516 (3)	C45—H45	0.93
C6—H6	0.98	C46—H46	0.93
C7—O1	1.216 (2)	C61—C62	1.382 (3)
C11—C16	1.381 (3)	C61—C66	1.386 (3)
C11—C12	1.383 (3)	C62—C63	1.379 (3)
C12—C13	1.383 (3)	C62—H62	0.93
C12—H12	0.93	C63—C64	1.356 (3)
C13—C14	1.361 (3)	C63—H63	0.93
C13—H13	0.93	C64—F4	1.359 (2)
C14—C15	1.356 (3)	C64—C65	1.361 (3)
C14—F1	1.360 (2)	C65—C66	1.386 (3)
C15—C16	1.383 (3)	C65—H65	0.93
C15—H15	0.93	C66—H66	0.93
C16—H16	0.93	N1—H2A	0.89 (2)
C31—C36	1.388 (3)	N2—H1A	0.86 (2)
C31—C32	1.390 (3)		
			
N1—C1—C11	109.51 (15)	C32—C31—C3	123.93 (17)
N1—C1—C2	107.55 (15)	C33—C32—C31	120.76 (19)
C11—C1—C2	112.45 (15)	C33—C32—H32	119.6
N1—C1—H1	109.1	C31—C32—H32	119.6
C11—C1—H1	109.1	C34—C33—C32	118.8 (2)
C2—C1—H1	109.1	C34—C33—H33	120.6
C7—C2—C1	109.37 (15)	C32—C33—H33	120.6
C7—C2—C3	105.39 (14)	F2—C34—C33	119.0 (2)
C1—C2—C3	112.61 (15)	F2—C34—C35	118.6 (2)
C7—C2—H2	109.8	C33—C34—C35	122.42 (19)
C1—C2—H2	109.8	C34—C35—C36	118.1 (2)
C3—C2—H2	109.8	C34—C35—H35	121.0
N2—C3—C31	113.52 (16)	C36—C35—H35	121.0
N2—C3—C2	107.60 (14)	C35—C36—C31	121.9 (2)
C31—C3—C2	111.73 (14)	C35—C36—H36	119.0
N2—C3—H3	107.9	C31—C36—H36	119.0
C31—C3—H3	107.9	C42—C41—C46	118.01 (18)
C2—C3—H3	107.9	C42—C41—C4	118.32 (17)
N2—C4—C41	113.15 (16)	C46—C41—C4	123.66 (16)
N2—C4—C5	108.19 (14)	C43—C42—C41	122.0 (2)
C41—C4—C5	112.21 (15)	C43—C42—H42	119.0
N2—C4—H4	107.7	C41—C42—H42	119.0
C41—C4—H4	107.7	C44—C43—C42	117.9 (2)
C5—C4—H4	107.7	C44—C43—H43	121.0
C7—C5—C6	108.66 (14)	C42—C43—H43	121.0
C7—C5—C4	106.05 (14)	C45—C44—F3	118.8 (2)
C6—C5—C4	113.94 (15)	C45—C44—C43	122.6 (2)
C7—C5—H5	109.4	F3—C44—C43	118.6 (2)
C6—C5—H5	109.4	C44—C45—C46	119.0 (2)
C4—C5—H5	109.4	C44—C45—H45	120.5
N1—C6—C61	109.65 (16)	C46—C45—H45	120.5
N1—C6—C5	107.95 (15)	C45—C46—C41	120.48 (19)
C61—C6—C5	110.70 (15)	C45—C46—H46	119.8
N1—C6—H6	109.5	C41—C46—H46	119.8
C61—C6—H6	109.5	C62—C61—C66	117.84 (19)
C5—C6—H6	109.5	C62—C61—C6	121.58 (18)
O1—C7—C2	124.21 (18)	C66—C61—C6	120.48 (18)
O1—C7—C5	123.78 (18)	C63—C62—C61	121.3 (2)
C2—C7—C5	111.69 (17)	C63—C62—H62	119.3
C16—C11—C12	117.76 (18)	C61—C62—H62	119.3
C16—C11—C1	122.54 (18)	C64—C63—C62	118.8 (2)
C12—C11—C1	119.69 (19)	C64—C63—H63	120.6
C13—C12—C11	121.3 (2)	C62—C63—H63	120.6
C13—C12—H12	119.3	C63—C64—F4	118.7 (2)
C11—C12—H12	119.3	C63—C64—C65	122.4 (2)
C14—C13—C12	118.5 (2)	F4—C64—C65	118.9 (2)
C14—C13—H13	120.7	C64—C65—C66	118.3 (2)
C12—C13—H13	120.7	C64—C65—H65	120.8
C15—C14—F1	118.8 (2)	C66—C65—H65	120.8
C15—C14—C13	122.4 (2)	C65—C66—C61	121.2 (2)
F1—C14—C13	118.8 (2)	C65—C66—H66	119.4
C14—C15—C16	118.5 (2)	C61—C66—H66	119.4
C14—C15—H15	120.8	C6—N1—C1	114.91 (15)
C16—C15—H15	120.8	C6—N1—H2A	107.3 (13)
C11—C16—C15	121.5 (2)	C1—N1—H2A	110.8 (13)
C11—C16—H16	119.2	C4—N2—C3	111.62 (15)
C15—C16—H16	119.2	C4—N2—H1A	111.2 (14)
C36—C31—C32	117.96 (18)	C3—N2—H1A	109.6 (14)
C36—C31—C3	118.09 (18)		
			
N1—C1—C2—C7	−1.1 (2)	C31—C32—C33—C34	−0.6 (3)
C11—C1—C2—C7	−121.79 (18)	C32—C33—C34—F2	−178.90 (18)
N1—C1—C2—C3	−117.95 (17)	C32—C33—C34—C35	−0.2 (3)
C11—C1—C2—C3	121.40 (17)	F2—C34—C35—C36	179.10 (18)
C7—C2—C3—N2	−61.43 (18)	C33—C34—C35—C36	0.4 (3)
C1—C2—C3—N2	57.74 (18)	C34—C35—C36—C31	0.3 (3)
C7—C2—C3—C31	173.32 (15)	C32—C31—C36—C35	−1.1 (3)
C1—C2—C3—C31	−67.52 (19)	C3—C31—C36—C35	177.44 (18)
N2—C4—C5—C7	59.39 (18)	N2—C4—C41—C42	−161.32 (17)
C41—C4—C5—C7	−175.09 (15)	C5—C4—C41—C42	75.9 (2)
N2—C4—C5—C6	−60.09 (19)	N2—C4—C41—C46	19.6 (2)
C41—C4—C5—C6	65.43 (19)	C5—C4—C41—C46	−103.2 (2)
C7—C5—C6—N1	−3.4 (2)	C46—C41—C42—C43	0.1 (3)
C4—C5—C6—N1	114.62 (17)	C4—C41—C42—C43	−179.00 (19)
C7—C5—C6—C61	116.65 (18)	C41—C42—C43—C44	−0.3 (3)
C4—C5—C6—C61	−125.36 (17)	C42—C43—C44—C45	0.1 (3)
C1—C2—C7—O1	128.28 (19)	C42—C43—C44—F3	−178.32 (19)
C3—C2—C7—O1	−110.4 (2)	F3—C44—C45—C46	178.73 (19)
C1—C2—C7—C5	−58.06 (19)	C43—C44—C45—C46	0.3 (3)
C3—C2—C7—C5	63.24 (18)	C44—C45—C46—C41	−0.5 (3)
C6—C5—C7—O1	−125.73 (19)	C42—C41—C46—C45	0.3 (3)
C4—C5—C7—O1	111.4 (2)	C4—C41—C46—C45	179.37 (19)
C6—C5—C7—C2	60.57 (19)	N1—C6—C61—C62	20.6 (3)
C4—C5—C7—C2	−62.32 (17)	C5—C6—C61—C62	−98.4 (2)
N1—C1—C11—C16	−28.4 (3)	N1—C6—C61—C66	−163.06 (17)
C2—C1—C11—C16	91.1 (2)	C5—C6—C61—C66	77.9 (2)
N1—C1—C11—C12	150.81 (19)	C66—C61—C62—C63	−0.6 (3)
C2—C1—C11—C12	−89.7 (2)	C6—C61—C62—C63	175.7 (2)
C16—C11—C12—C13	0.6 (3)	C61—C62—C63—C64	−0.4 (4)
C1—C11—C12—C13	−178.6 (2)	C62—C63—C64—F4	−179.1 (2)
C11—C12—C13—C14	0.4 (4)	C62—C63—C64—C65	1.3 (4)
C12—C13—C14—C15	−1.2 (4)	C63—C64—C65—C66	−1.0 (3)
C12—C13—C14—F1	178.0 (2)	F4—C64—C65—C66	179.5 (2)
F1—C14—C15—C16	−178.3 (2)	C64—C65—C66—C61	−0.2 (3)
C13—C14—C15—C16	0.8 (4)	C62—C61—C66—C65	1.0 (3)
C12—C11—C16—C15	−1.0 (3)	C6—C61—C66—C65	−175.47 (18)
C1—C11—C16—C15	178.22 (19)	C61—C6—N1—C1	−179.30 (16)
C14—C15—C16—C11	0.3 (3)	C5—C6—N1—C1	−58.6 (2)
N2—C3—C31—C36	159.61 (16)	C11—C1—N1—C6	−176.23 (16)
C2—C3—C31—C36	−78.5 (2)	C2—C1—N1—C6	61.3 (2)
N2—C3—C31—C32	−21.9 (2)	C41—C4—N2—C3	171.16 (15)
C2—C3—C31—C32	100.0 (2)	C5—C4—N2—C3	−63.87 (18)
C36—C31—C32—C33	1.3 (3)	C31—C3—N2—C4	−170.94 (14)
C3—C31—C32—C33	−177.17 (18)	C2—C3—N2—C4	64.88 (18)

### Hydrogen-bond geometry (Å, °)

**Table:**

*D*—H···*A*	*D*—H	H···*A*	*D*···*A*	*D*—H···*A*
C15—H15···F4^i^	0.93	2.52	3.254 (3)	136
C3—H3···O1^ii^	0.98	2.56	3.358 (2)	138
N2—H1A···O1^iii^	0.86 (2)	2.53 (2)	3.292 (2)	148 (3)
N1—H2A···Cg3^iv^	0.89 (2)	2.70 (3)	3.549 (3)	160 (2)
C36—H36···Cg2^v^	0.93	2.81	3.696 (3)	160
C42—H42···Cg1^v^	0.93	2.78	3.651 (3)	157
C45—H45···Cg3^iii^	0.93	2.65	3.494 (3)	151

Symmetry codes: (i) −*x*, −*y*+2, −*z*; (ii) −*x*+1/2, *y*−1/2, −*z*+1/2; (iii) *x*, *y*−1, *z*; (iv) −*x*, −*y*+1, −*z*; (v) −*x*+1/2, *y*+1/2, −*z*+1/2.
